# Neutrophil-to-lymphocyte ratio is associated with 28-day mortality in patients with severe fever with thrombocytopenia syndrome

**DOI:** 10.1186/s12879-022-07206-8

**Published:** 2022-03-06

**Authors:** Yun Liu, Jun Ni, Yali Xiong, Chao Wu, Fei He

**Affiliations:** 1 Department of Emergency Medicine, Nanjing Drum Tower Hospital, The Affiliated Hospital of Nanjing University Medical School, Nanjing, 210008 China; 2 Department of Clinical Laboratory, Nanjing Drum Tower Hospital, The Affiliated Hospital of Nanjing University Medical School, Nanjing, 210008 China; 3 Department of Infectious Disease, Nanjing Drum Tower Hospital, The Affiliated Hospital of Nanjing University Medical School, Nanjing, 210008 China

**Keywords:** Neutrophil-to-lymphocyte ratio, Mortality, Severe fever with thrombocytopenia syndrome

## Abstract

**Objectives:**

To determine the association of the neutrophil-to-lymphocyte ratio (NLR) with 28-day mortality in patients with severe fever with thrombocytopenia syndrome (SFTS).

**Methods:**

A single-centre retrospective analysis was performed in an emergency department from January 01, 2018, to June 30, 2021. Univariate and multivariable Cox proportional hazards regression models were used to investigate the prognostic factors associated with 28-day mortality. Kaplan–Meier curves were analysed in patients stratified by the optimal cut-off point of the NLR determined using a receiver operating characteristic (ROC) curve.

**Results:**

In total, 182 SFTS patients were included, and 24 (13.2%) died within 28 days. The median age of the included patients was 59.64 ± 12.74 years, and 48.4% (88/182) were male. The patients in the non-survival group had significantly higher NLRs than those in the survival group (6.91 ± 6.73 vs. 2.23 ± 1.83). The NLR was a significant predictor of 28-day mortality (adjusted HR: 1.121, 95% CI: 1.033, 1.215). The area under the ROC curve of the NLR for predicting 28-day mortality was 0.743 (95% CI: 0.624, 0.862), and the optimal cut-off value was 4.19 (sensitivity, 54.2%; specificity, 89.2%). In addition, 28-day mortality in the patients with an NLR ≥ 4.19 was notably higher than that in the patients with an NLR < 4.19 (43.3% vs. 7.2%), and Kaplan–Meier analysis showed that the patients with an NLR ≥ 4.19 had a significantly lower survival rate than those with an NLR < 4.19.

**Conclusions:**

The NLR was a significant, independent predictor of 28-day mortality in SFTS patients.

**Supplementary Information:**

The online version contains supplementary material available at 10.1186/s12879-022-07206-8.

## Introduction

Severe fever with thrombocytopenia syndrome (SFTS) is an emerging infectious disease worldwide characterized by fever, leukopenia, thrombocytopenia, malaise, headache, myalgia, and dizziness [1]. SFTS is caused by the SFTS virus, a newly identified bunyavirus that appears to be carried by ticks [2]. The mortality rate of SFTS patients is high because of the rapid development of multiple organ failure, and no effective therapy strategy has been established to date [1]. Therefore, the exploration of possible predictors for identifying patients with a higher risk of death due to SFTS may help guide early management strategies to improve prognosis. Recent studies have revealed that some new scoring models consisting of certain clinical parameters can be used to efficiently predict the prognosis of SFTS patients [3, 4]. However, it is difficult to obtain and evaluate the parameters in these models, and the models are excessively complicated to use in clinical practice.

The neutrophil-to-lymphocyte ratio (NLR) is a biomarker in peripheral blood that reflects systemic inflammation and immunity [5]. Recently, emerging evidence has shown that a higher NLR is associated with a higher risk of mortality in patients with acute medical conditions, such as sepsis [6], acute pancreatitis [7] and cerebral haemorrhage [8]. Nevertheless, few studies have investigated the relationship between the NLR and mortality in SFTS patients. Therefore, the aim of this study was to evaluate the association of the NLR with the prognosis in SFTS patients. We speculated that a higher NLR was a novel biomarker indicating a poor prognosis in patients with SFTS.

## Method

### Patients and selection criteria

This was a single-centre retrospective study that included 182 patients with SFTS treated in the Emergency Department of Nanjing Drum Tower Hospital from January 01, 2018, to June 30, 2021. The inclusion criteria included adult patients who met the diagnostic criteria for SFTS [1] and had complete clinical data. Patients were excluded if the following criteria were met: (1) age under 18 years or (2) a history of haematologic disorder. Blood samples were obtained by peripheral vein puncture from each patient during the hospital stay in the emergency department.

### Data collection

The following demographic characteristics and clinical data were extracted from the electronic medical record system of our institution: (1) the baseline demographic and clinical characteristics were age, sex, coexisting conditions [hypertension (HTN), diabetes mellitus (DM), chronic obstructive pulmonary disease (COPD) and coronary artery disease (CAD)], fever, vomiting, diarrhoea, headache, abdominal pain, cough lymphadenopathy, petechiae, and Glasgow coma scale (GCS) scores), and (2) the laboratory data included the white blood cell count (WBC count, normal reference range of 3.5–9.5 × 10^9^/L), absolute neutrophil count (ANC, normal reference range of 1.6–6.3 × 10^9^/L), absolute lymphocyte count (ALC, normal reference range of 1.1–3.2 × 10^9^/L), neutrophil to lymphocyte ratio (NLR, calculated by dividing the neutrophil count by the lymphocyte count), red cell distribution width (RDW, normal reference range of 0–14%), platelet count (PLT count, normal reference range of 125–350 × 10^9^/L), prothrombin time (PT, normal reference range of 10-15s), activated partial thromboplastin time (APTT, normal reference range of 25–31.3 s), and levels of alanine aminotransferase (ALT, normal reference range of 13–69 U/L), aspartate aminotransferase (AST, normal reference range of 15–46 U/L), lactate dehydrogenase (LDH, normal reference range of 313–618 U/L), total bilirubin (TBil, normal reference range of 5.1–28 μmol/L), direct bilirubin in serum (DBil, normal reference range of 0–10 μmol/L), serum creatinine (SCr, normal reference range or 58–110 μmol/L), and blood urea nitrogen (BUN, normal reference range of 3.2–7.1 mmol/L). Multiple organ dysfunction syndrome (MODS) was defined as progressive physiological dysfunction or failure occurring in more than two organ systems simultaneously or sequentially because of the severe medical condition (such as serious infection) [9]. The primary outcome was 28-day mortality. Moreover, we followed them up by a telephone interview if the patients were discharged within 28 days. An Excel file was used to store the clinical and laboratory data.

### Statistical analysis

All the data in the present study were analysed using SPSS 22.0 for Windows (SPSS Inc., Chicago, IL, USA). Normally distributed continuous variables are presented as the mean ± standard deviation (SD) and were compared using Student’s t test. Non-normally distributed continuous data are presented as the median with interquartile range (IQR) and were compared using the Mann–Whitney U test. Categorical data are presented as frequencies and percentages and were compared using Fisher’s exact test or the chi-square test where appropriate. The associations of the WBC count, ANC, ALC and NLR with the 28-day outcome were determined using univariate and multivariable Cox regression analyses. The receiver operating characteristic (ROC) curve test was applied to evaluate the ability of the WBC count, ANC and NLR to predict the 28-day outcome, and the optimal cut-off values of the WBC count, ANC and NLR were determined by the maximum Youden index. Survival curves were estimated using the Kaplan–Meier method, and the mortality of each group of patients was compared with the log-rank test. A *P* value of < 0.05 was considered statistically significant.

## Results

**Fig. 1:**
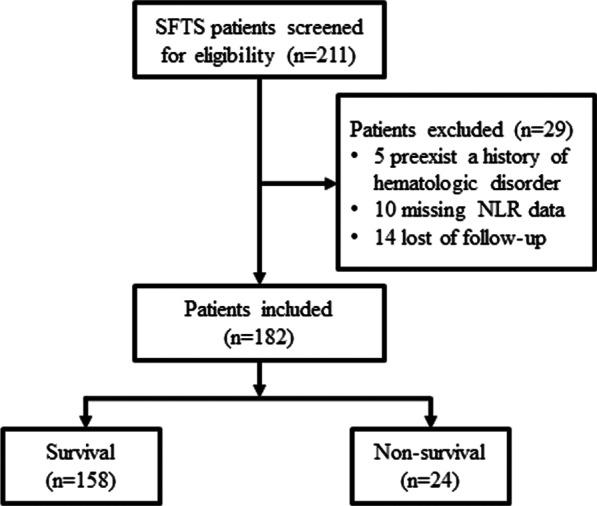
Flow chart of the study participants

**Fig. 2:**
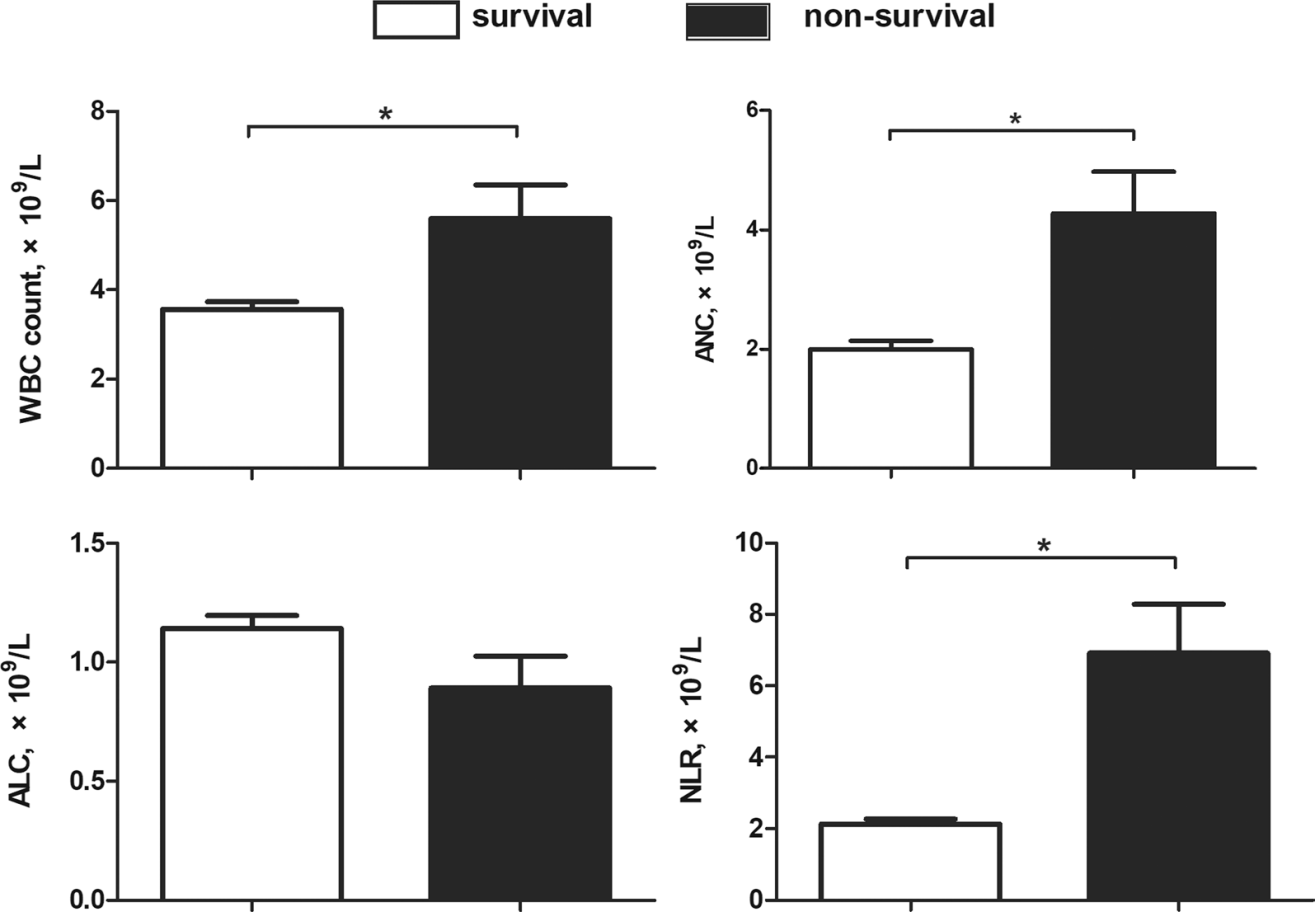
Comparison of the WBC count, ANC, ALC and NLR between the survival and non-survival groups (*P < 0.05). *ANC* absolute neutrophil count, *ALC* absolute lymphocyte count, *NLR* neutrophil-to-lymphocyte ratio, *WBC* white blood cell

**Fig. 3:**
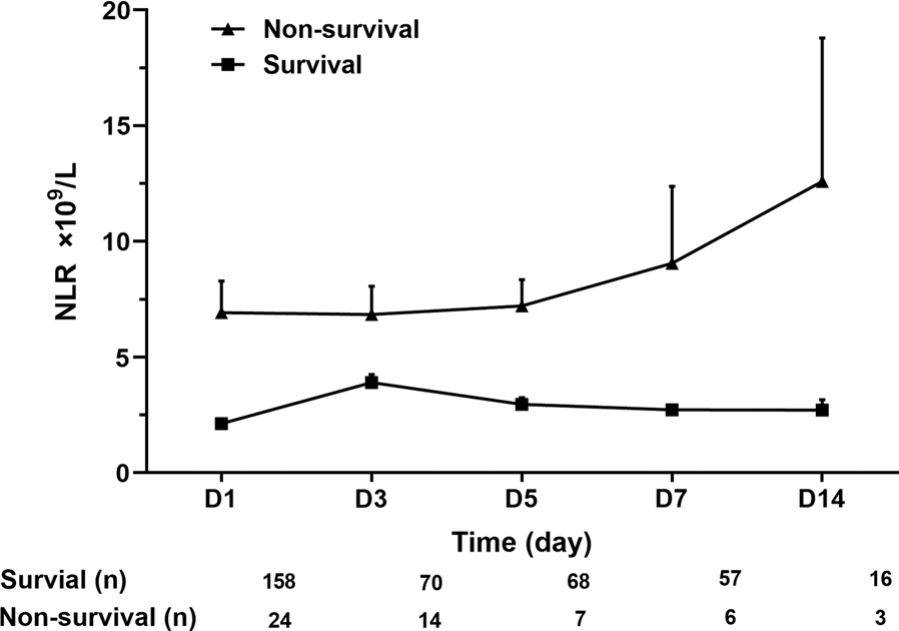
Changes in the mean (± SE) NLR over 14 days in patients in the survival and non-survival groups. *SE* standard error, *NLR* neutrophil-to-lymphocyte ratio

In the present study, 211 patients diagnosed with SFTS were screened for eligibility, and 182 met the inclusion criteria (Fig. 1). The median age of the SFTS patients was 59.64 ± 12.74 years, and 48.4% (88/182) were male. Overall, the 28-day mortality was 13.2% (24/182), and the length of stay in the hospital was 9.00 days (7.00, 12.00). A comparison of the baseline clinical characteristics and laboratory findings between the patients in the survival and non-survival groups is shown in Table 1. The patients in the non-survival group showed significantly higher baseline demographic and laboratory data values, including age, APTT, and levels of AST, LDH, SCr and BUN, than those in the survival group. Moreover, the proportion of MODS in the non-survival group was notably higher than that in the survival group. However, the GCS score and PLT count in the non-survival group were notably lower than those in the survival group. Moreover, the length of hospital stay in the non-survival group was also notably shorter than that in the survival group. Additionally, the patients in the survival group had a significantly lower WBC count, ANC, and NLR than those in the non-survival group (Fig. 2; Table 1). As shown in Fig. 3, the NLR in the non-survival group gradually increased after admission and reached its peak level at 14 days, whereas the NLR in the survival group peaked within 3 days after admission and then gradually decreased within 3–14 days. The NLR over 14 days was consistently higher in the non-survival group patients than in the survival group patients.

**Table 1:** Baseline clinical characteristics and laboratory parameters according to the 28-day outcome

Variable	Survival (n = 158)	Non-survival (n = 24)	*P* Value
Demographics			
Age, years	58.32 ± 12.36	68.29 ± 12.00	0.001
Male, sex, n (%)	79 (50.0)	9 (37.5)	0.254
Chronic comorbidities			
HTN, n (%)	33 (20.9)	9 (37.5)	0.072
DM, n (%)	9 (5.7)	4 (16.7)	0.052
COPD, n (%)	1 (0.6)	1 (4.2)	0.620
CAD, n (%)	1 (0.6)	1 (4.2)	0.620
Clinical manifestations			
Fever, n (%)	156 (98.7)	23 (95.8)	0.857
Vomiting, n (%)	29 (18.4)	7 (29.2)	0.215
Diarrhea, n (%)	40 (25.3)	7 (29.2)	0.688
Headache, n (%)	56 (35.4)	6 (25.0)	0.315
Abdominal pain, n (%)	17 (10.8)	1 (4.2)	0.521
Cough, n (%)	27 (17.1)	3 (12.5)	0.788
Lymphadenop athy, n (%)	62 (39.2)	6 (25.0)	0.179
Petechiae, n (%)	22 (13.9)	6 (25.0)	0.161
GCS scores, mean ± SD	13.88 ± 2.12	9.50 ± 2.75	0.001
Laboratory parameters			
WBC count, mean ± SD, ×10^9^/L	3.57 ± 2.12	5.06 ± 3.69	0.014
ANC, mean ± SD, ×10^9^/L	2.00 ± 1.71	4.28 ± 3.42	0.004
ALC, mean ± SD, ×10^9^/L	1.14 ± 0.69	0.89 ± 0.66	0.097
NLR, mean ± SD, ×10^9^/L	2.13 ± 1.83	6.91 ± 6.73	0.002
RDW, mean ± SD, %	13.16 ± 0.86	14.00 ± 2.28	0.087
PLT count, median (IQR), ×10^9^/L	57.5 (37.50, 86.00)	35.50 (25.00, 48.25)	0.001
PT, mean ± SD, s	11.30 ± 1.02	12.64 ± 3.02	0.051
APTT, mean ± SD, s	36.60 ± 9.56	59.31 ± 17.09	0.001
ALT, median (IQR), U/L	72.05 (48.53, 121.40)	62.00 (42.70, 85.80)	0.241
AST, median (IQR), U/L	112.00 (66.95, 232.25)	241.70 (129.13, 547.03)	0.001
LDH, median (IQR), U/L	717.50 (422.5, 1221.00)	2110.50 (1109.75, 3575.00)	0.001
TBil, median (IQR), μmol/L	10.10 (6.95, 16.85)	8.10 (5.90, 15.30)	0.295
DBil, median (IQR), μmol/L	4.20 (2.55, 6.25)	4.20 (2.45, 9.25)	0.846
SCr, mean ± SD, μmol/L	59.78 ± 36.11	101.79 ± 60.35	0.003
BUN, median (IQR), mmol/L	3.60 (2.75, 4.70)	5.80 (4.63, 8.46)	0.001
Complications			
MODS, n (%)	28 (17.7)	23 (95.8)	0.001
Duration of hospital			
Length of hospital stay, median (IQR) days	10.00 (7.00,12.25)	5.50 (2.00, 11.25)	0.001

*ANC* absolute neutrophil count, *ALC* absolute lymphocyte count, *AST* aspartate aminotransferase, *ALT* alanine aminotransferase, *APTT* activated partial thromboplastin time, *BUN* blood urea nitrogen, *CI* confidence interval, *COPD* chronic obstructive pulmonary disease, *CAD* coronary artery disease, *DBil* direct bilirubin, *DIC* disseminated intravascular coagulation, *DM* diabetes mellitus, *GCS* glasgow coma scale, *HTN* hypertension, *LDH* lactate dehydrogenase, *MODS* multiple organ dysfunction syndrome, *NLR* neutrophil-to-lymphocyte ratio, *OR* odds ratio, *PLT* platelet, *PT* prothrombin time, *RDW* red cell volume distribution width, *SCr* serum creatinine, *TBil* total bilirubin, *UA* uric acid, *WBC* white blood cell

**Fig. 4:**
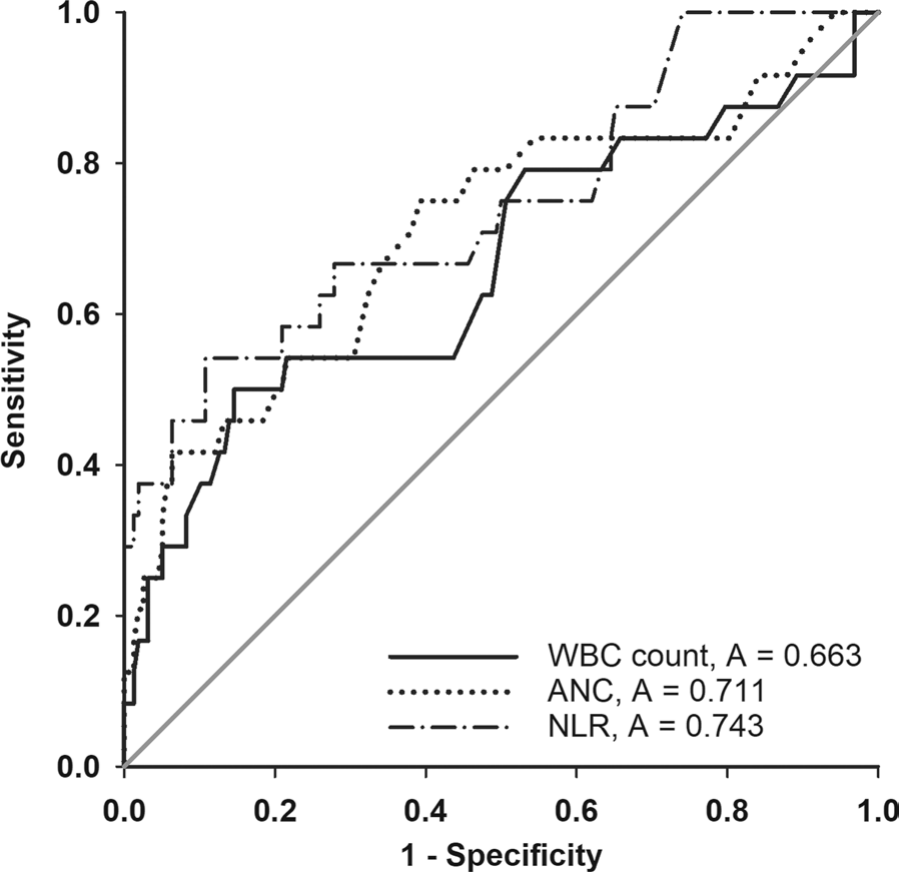
ROC curve analysis of the WBC count, ANC and NLR for 28-day mortality in SFTS patients. ANC, absolute neutrophil count; NLR, neutrophil-to-lymphocyte ratio; ROC, receiver operating characteristic; SFTS, severe fever with thrombocytopenia syndrome; WBC, white blood cell

The association of leukocyte counts and the NLR with the 28-day mortality in the SFTS patients was further investigated via univariate and multivariable Cox regression analyses. As shown in Table 2, our results indicated that the WBC count [adjusted hazard ratio (HR): 1.264, 95% CI: 1.030, 1.550, *P* = 0.025], ANC (adjusted HR: 1.385, 95% CI: 1.117, 1.717, *P* = 0.003) and NLR (adjusted HR: 1.121, 95% CI: 1.033, 1.215, *P* = 0.006) were potential predictors independently associated with the 28-day mortality after adjustment for confounders (Additional file 1: Table S1). ROC curves of the 28-day mortality of the SFTS patients generated using the independent predictors (WBC count, ANC and NLR) are plotted in Fig. 4. As shown in Table 3, the AUCs of the WBC count, ANC and NLR were 0.663 (95% CI: 0.527, 0.799, *P* = 0.01), 0.711 (95% CI: 0.583, 0.838, *P* = 0.001) and 0.743 (95% CI: 0.624, 0.862, *P* = 0.001), respectively. When the optimal cut-off value (maximum Youden index) was 4.19, the sensitivity and specificity of the NLR for the 28-day mortality in the SFTS patients were 0.542 and 0.892, respectively. Meanwhile, the positive likelihood ratio (LR+) was 5.034, and the negative likelihood ratio (LR−) was 0.514.

**Table 2:** Independent predictors associated with the 28-day mortality in SFTS patients by univariate and multivariable Cox regression analysis

Independent variable	Unadjusted		Adjusted^a^	
	HR (95%CI)	P Value	HR (95%CI)	P Value
WBC count, ×10^9^/L	1.263 (1.120, 1.424)	0.001	1.264 (1.030, 1.550)	0.025
ANC×10^9^/L	1.326 (1.181, 1.489)	0.001	1.385 (1.117, 1.717)	0.003
ALC×10^9^/L	0.551 (0.265, 1.143)	0.109	0.559 (0.230, 1.362)	0.201
NLR	1.201 (1.134, 1.272)	0.001	1.121 (1.033, 1.215)	0.006

*ANC* absolute neutrophil count, *ALC* absolute lymphocyte count, *CI* confidence interval, *HR* hazard ratio, *NLR* neutrophil-to-lymphocyte ratio, *WBC* white blood cell

^a^Adjustment by age, sex, GCS scores, PLT count, APTT, AST, LDH, SCr, BUN

**Table 3:** Prediction analysis of WBC, ANC and NLR of the 28-day mortality

Variable	AUC	95%CI	Cut-off value	Sensitivity	Specificity	LR+	LR−	P Value
WBC count	0.663	0.527, 0.799	5.55	0.500	0.854	3.434	0.585	0.01
ANC	0.711	0.583, 0.838	1.85	0.750	0.608	1.911	0.412	0.001
NLR	0.743	0.624, 0.862	4.19	0.542	0.892	5.034	0.514	0.001

*AUC* area under curve,* CI* confidence interval,* LR+* likelihood ratio positive,* LR-* likelihood ratio negative,* ANC* absolute neutrophil count,* NLR* neutrophil-to-lymphocyte ratio,* WBC* white blood cell,* YI* Youden index

**Fig. 5:**
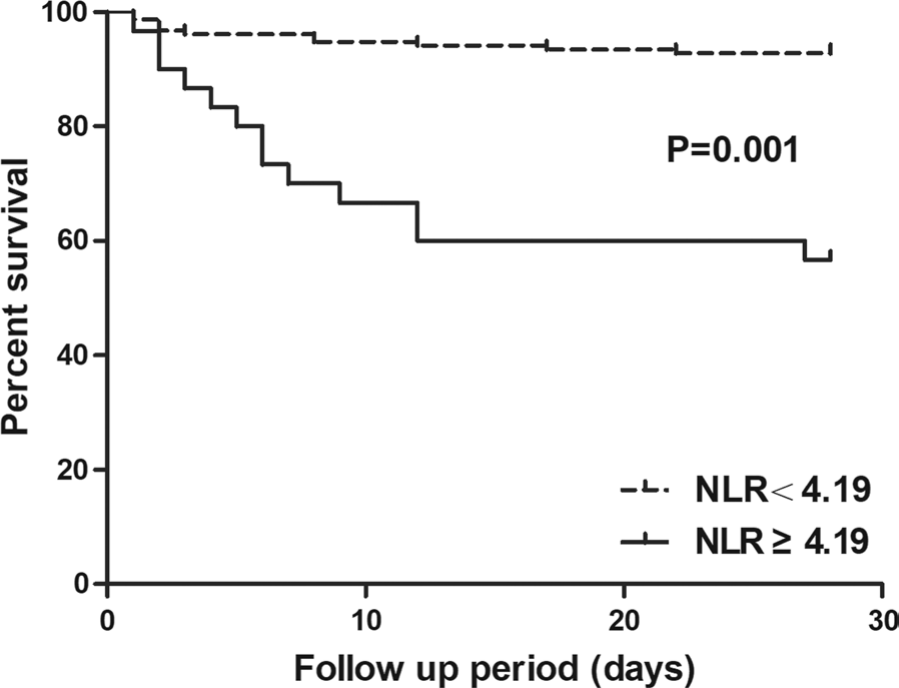
Kaplan–Meier survival curve of 28-day mortality according to the optimal cut-off of NLR = 4.19

To further explore the predictive value of the NLR for short-term outcomes, we divided the SFTS patients into two groups according to the optimal cut-off value of NLR = 4.19. As shown in Table 4, significant differences appeared in the GCS scores, BUN level, frequency of MODS and length of hospital stay between the two groups. Moreover, the 28-day mortality in the NLR ≥ 4.19 group was notably higher than that in the NLR < 4.19 group. Additionally, Kaplan–Meier analysis showed that the patients with an NLR ≥ 4.19 had a significantly lower chance of survival than the patients with an NLR < 4.19 (log rank *P* = 0.001, Fig. 5).

**Table 4:** Baseline clinical characteristics and laboratory parameters by NLR < 4.19 and NLR ≥ 4.19 groups

Variable	NLR ≥ 4.19 (n = 30)	NLR < 4.19 (n = 152)	*P* Value
Demographics			
Age, years	62.97 ± 13.43	58.98 ± 12.54	0.118
Male, sex, n (%)	17 (56.7)	71 (46.7)	0.319
Chronic comorbidities			
HTN, n (%)	9 (30.0)	33 (21.7)	0.325
DM, n (%)	2 (6.7)	11 (7.2)	0.912
COPD, n (%)	1 (3.3)	1 (7.0)	0.744
CAD, n (%)	1 (3.3)	1 (7.0)	0.744
Clinical manifestations			
Fever, n (%)	29 (96.7)	150 (98.7)	0.993
Vomiting, n (%)	8 (26.7)	28 (18.4)	0.300
Diarrhea, n (%)	5 (16.7)	42 (27.6)	0.210
Headache, n (%)	10 (33.3)	52 (34.2)	0.926
Abdominal pain, n (%)	2 (6.7)	16 (10.5)	0.755
Cough, n (%)	7 (23.3)	23 (15.1)	0.269
Lymphadenopathy, n (%)	12 (40.0)	56 (36.8)	0.744
Petechiae, n (%)	7 (23.3)	21 (13.8)	0.187
GCS scores, mean ± SD	11.76 ± 3.26	13.59 ± 2.43	0.006
Laboratory parameters			
RDW, mean ± SD, %	13.23 ± 0.93	13.28 ± 1.22	0.840
PLT count, median (IQR), ×10^9^/L	55.00 (36.00, 88.50)	48.50 (34.00, 77.50)	0.327
PT, mean ± SD, s	12.35 ± 2.73	11.30 ± 1.06	0.059
APTT, mean ± SD, s	43.07 ± 15.66	39.81 ± 12.57	0.121
ALT, median (IQR), U/L	62.00 (36.98, 87.60)	73.30 (47.30, 123.50)	0.093
AST, median (IQR), U/L	98.75 (73.90, 220.63)	132.60 (71.7, 262.90)	0.534
LDH, median (IQR), U/L	755.00 (548.25, 1417.25)	747.50 (399.5,1282.25)	0.668
TBil, median (IQR), μmol/L	10.00 (6.65, 24.45)	10.00 (6.90,16.40)	0.425
DBil, median (IQR), μmol/L	4.45 (2.43, 10.78)	4.20 (2.50, 6.05)	0.243
SCr, mean ± SD, μmol/L	80.72 ± 65.32	62.31 ± 35.75	0.144
BUN, median (IQR), mmol/L	4.70 (3.85,6.30)	3.50 (2.70, 4.90)	0.002
Complications			
MODS, n (%)	16 (53.3)	35 (23.0)	0.001
Duration of hospital			
Length of hospital stay, median (IQR) days			
Outcome	7.00 (5.75, 12.00)	9.50 (7.00, 12.00)	0.047
28-day mortality, n (%)	13 (43.3)	11 (7.2)	0.001

*BUN* blood urea nitrogen, *COPD* chronic obstructive pulmonary disease, *CAD* coronary artery disease, *DIC* disseminated intravascular coagulation, *DM* diabetes mellitus, *GCS* Glasgow coma scale, *HTN* hypertension, *MODS* multiple organ dysfunction syndrome, *SCr* serum creatinine, *UA* uric acid, *RDW* red cell volume distribution width, *PLT* platelet, *APTT* activated partial thromboplastin time, *PT* prothrombin time, *AST* aspartate aminotransferase, *ALT* alanine aminotransferase, *LDH* lactate dehydrogenase, *TBil* total bilirubin, *DBil* direct bilirubin

## Discussion

In the present study, the findings showed that the NLR was a significant, independent predictor of 28-day mortality in SFTS patients. Moreover, when the SFTS patients were divided into two groups based on the optimal cut-off value of NLR = 4.19, the patients with an increased NLR had a significantly lower chance of survival at 28 days. Thus, physicians should pay attention to this group of SFTS patients with an increased NLR, and management strategies are required in a timely manner to improve the outcome in these patients.

The NLR is a widely used biomarker of cellular immune activation for the assessment of severity and prognosis in various infectious and non-infectious diseases [5, 6, 10]. Moreover, the NLR may serve as a simple haematological parameter for discriminating between bacterial and viral infections since the NLR in bacterial infections is usually higher than that in viral infections [11]. The NLR improves clinicians’ understanding of systemic inflammation, the pathophysiology of the cellular immune response, and the interaction between innate and adaptive immunity as well as the clinical consequences of disease [12]. In the present study, the results showed that the SFTS patients in the non-survival group had higher neutrophil counts and lower lymphocyte counts than those in the survival group. Moreover, we also found that the patients with an increased NLR had a significantly higher incidence of MODS and a lower chance of survival within 28 days, which is consistent with the latest study by Wang X et al. [13]. Therefore, our findings further extend the evidence of the NLR as a prognostic predictor, demonstrating an increased risk of short-term mortality in SFTS patients. Of note, Wang X et al. [13] found that the NLR at discharge in surviving patients was significantly lower than that at admission, whereas the NLR at the time of death in non-surviving patients was higher than that at admission. Similar findings were demonstrated in our study: patients in the non-survival group showed gradually increasing NLRs during the hospital stay, whereas the NLR in the patients in the survival group peaked within 3 days after admission and then gradually decreased. Moreover, the NLR was consistently higher in the non-survival group patients than in the survival group patients during the hospital stay. Therefore, the dynamic change in the NLR not only reflects the improvement or deterioration in the clinical status associated with the SFTS virus infection but also helps clinicians assess the severity of disease and make treatment decisions.

Currently, the underlying mechanism between increased NLRs and increased mortality in SFTS patients remains unclear. In the early phase, the SFTS virus invades patients and triggers the release of pro-inflammatory cytokines. Then, neutrophils are dispatched to the site of inflammation, providing rapid sensing and elimination of pathogens [14]. The tempered inflammatory response is the first line of host defence against SFTS virus attack. In the progression phase, the SFTS virus itself and the provoked inflammatory response damage the immune system of the host, manifested mainly by a significant decrease in lymphocyte levels, particularly in patients with severe conditions and non-survivors [15]. In the present study, the WBC count and ANC were higher in the non-survival group than in the survival group, whereas the ALC was lower in the non-survival group than in the survival group. Taken together, these findings might explain why an increased NLR was observed in the non-survival group in our study. In addition, we also found that the SFTS patients with an increased NLR showed a significantly higher incidence of MODS, which indicated the damaged immunity status and organ function of the hosts after SFTS virus infection, which may be responsible for the worse condition and prognosis of these patients.

Several limitations should be acknowledged in our study. First, this is a retrospective, single-centre study with a small sample size. Therefore, larger-scale, better-designed studies are needed to validate our findings. Second, due to limited clinical data, we did not include clinical parameters in the Cox regression analysis that were missed in half or more of the patients; these parameters included levels of procalcitonin (PCT), C-reactive protein (CRP), D-dimer, and viral load. Thus, potential selection bias is very likely to exist, and more accurate outcomes would result from adjustments in these potential confounders. Finally, we only determined the association of increased NLRs with short-term mortality in SFTS patients. Because the prognosis of the patients could change in the long term, the predictive value of the NLR in long-term mortality should be clarified in further study.

In summary, our study demonstrates that the NLR, an inexpensive, easily available haematological parameter, is associated with 28-day mortality in SFTS patients.

## Supplementary Information

**Additional file 1**: **Table S1. **Multivariate Cox regressionanalysis predicting the 28-day outcome. 

## Data Availability

The datasets used and/or analyzed during the current study are available from the corresponding author on reasonable request.
