# Supplementary material for: Neutrophil-to-lymphocyte ratio is associated with 28-day mortality in patients with severe fever with thrombocytopenia syndrome
Source: BMC Infect Dis. 2022 Mar 6;22:225. doi: 10.1186/s12879-022-07206-8 (PMC8898529; doi:10.1186/s12879-022-07206-8)
Supplement: Supplementary file 1 — Additional file 1: Table S1. Multivariate Cox regressionanalysis predicting the 28-day outcome. [file 12879_2022_7206_MOESM1_ESM.docx]

**Table S1** Multivariate Cox regression analysis predicting the 28-day outcome

| **Model** | **Independent variable** | | **Adjusted HR (95% CI)** | **P value** |
| --- | --- | --- | --- | --- |
| Model 1 (with WBC count) | Age | 1.090 (1.033,1.184) | | 0.013 |
|  | Sex | 0.840 (0.285,2.472) | | 0.751 |
|  | GCS scores | 0.742 (0.594, 0.927) | | 0.009 |
|  | PLT count | 0.961 (0.928, 0.996) | | 0.027 |
|  | APTT | 1.062 (1.027, 1.098) | | 0.001 |
|  | AST | 0.999 (0.998, 1.001) | | 0.272 |
|  | LDH | 1.000 (1.000,1.000) | | 0.632 |
|  | SCr | 1.012 (0.995, 1.028) | | 0.159 |
|  | BUN | 0.977 (0.838, 1.140) | | 0.770 |
|  | WBC count | 1.264 (1.030, 1.550) | | 0.025 |
| Model 2 (with ANC) | Age | 1.095 (1.018,1.177) | | 0.014 |
|  | Sex | 1.196 (0.387,3.961) | | 0.756 |
|  | GCS scores | 0.732(0.585,0.915) | | 0.006 |
|  | PLT count | 0.956 (0.920,0.993) | | 0.019 |
|  | APTT | 1.058 (1.024,1.002) | | 0.001 |
|  | AST | 0.999 (0.998,1.001) | | 0.403 |
|  | LDH | 1.000 (1.000,1.000) | | 0.916 |
|  | SCr | 1.013 (0.991,1.035) | | 0.265 |
|  | BUN | 0.964 (0.756, 1.228) | | 0.764 |
|  | ANC | 1.385 (1.117,1.717) | | 0.003 |
| Model 3 (with ALC) | Age | 1.088 (1.028,1.151) | | 0.003 |
|  | Sex | 0.896 (0.307,2.614) | | 0.840 |
|  | GCS scores | 0.664 (0.528,0.836) | | 0.001 |
|  | PLT count | 0.974 (0.948,1.002) | | 0.069 |
|  | APTT | 1.044 (1.009,1.080) | | 0.013 |
|  | AST | 0.999 (0.998,1.001) | | 0.238 |
|  | LDH | 1.000 (1.000,1.000) | | 0.108 |
|  | SCr | 1.016 (0.995,1.038) | | 0.139 |
|  | BUN | 0.967 (0.767,1.220) | | 0.778 |
|  | ALC | 0.559 (0.230,1.362) | | 0.201 |
| Model 4 (with NLR) | Age | 1.094 (1.020,1.173) | | 0.012 |
|  | Sex | 1.211 (0.374,3.920) | | 0.749 |
|  | GCS scores | 0.718 (0.570,0.904) | | 0.005 |
|  | PLT count | 0.966 (0.935,0.999) | | 0.043 |
|  | APTT | 1.038 (1.004,1.074) | | 0.029 |
|  | AST | 1.000 (0.998,1.001) | | 0.605 |
|  | LDH | 1.000 (1.000,1.000) | | 0.184 |
|  | SCr | 1.018 (0.994,1.043) | | 0.145 |
|  | BUN | 0.932 (0.712,1.220) | | 0.608 |
|  | NLR | 1.121 (1.033,1.215) | | 0.006 |

ANC, absolute neutrophil count; ALC, absolute lymphocyte count; BUN, blood urea nitrogen; GCS, glasgow coma scale; SCr, serum creatinine; PLT, platelet; APTT, activated partial thromboplastin time; PT, prothrombin time; AST, aspartate aminotransferase; LDH, lactate dehydrogenase; NLR, neutrophil-to-lymphocyte ratio; WBC, white blood cell; HR, hazard ratio; CI, confidence Interval
